# Generate vector graphics of fine-grained pattern based on the Xception edge detection

**DOI:** 10.1371/journal.pone.0318930

**Published:** 2025-06-11

**Authors:** Anqi Chen, Yicui Peng, Meng Li, Hao Chen, Chang Liu, Jinrong Hu, Xiang Wen, Guo Huang

**Affiliations:** 1 Chengdu Technological University, Chengdu, China; 2 School of Computer Science, Chengdu University of Information Technology, Chengdu, China; 3 College of Computer Science, Sichuan University, Chengdu, China; 4 China Mobile (Chengdu) Industry Research Institute, Chengdu, China; 5 Leshan Normal University, Leshan, China; Galgotias University, INDIA

## Abstract

Harnessing the power of artificial intelligence(AI) approaches to innovatively generating the vector graphics of fine-grained patterns has become an important task in image edge extraction, particularly on the domain of intangible cultural heritage (ICH) images where they are typically fine-grained and having the complex edges. With higher autonomy, the machine learning algorithms are able to accurately extract the image information, understand and convey the concept contained in it. In this paper, we take Qiang embroidery patterns as an example due to containing fine-grained patterns, which is more suitable for the study of image processing and pattern recognition techniques. We firstly adopt appropriate pre-processing methods, improved adaptive median filtering(IAMF) and non-local mean for the two different types of Qiang embroidery patterns to reduce image noise. Then, the Xception algorithm based on convolutional neural networks(CNNs) is used for edge detection and extraction to generate vector graphics of the patterns. Experimental results show that Qiang embroidery patterns, after denoising and edge extraction, can be clearly identified the shape characteristics of the patterns. Based on this approach, the images can be converted into vector graphics for the digital preservation and further artistic reinterpretation. The use of the Xception algorithm effectively solves the problem of extraction of Qiang embroidery in two-dimensional vectorial images. In addition, our proposed method provides a reliable practical reference for the preservation of other related ICH images.

## 1. Introduction

Intangible Cultural Heritage (referred to as “ICH” hereinafter) embodies the rich civilization of human society and constitutes an essential component of global cultural diversity. In the era of globalization, the preservation, inheritance, and development of ICH are facing unprecedented challenges, underscoring their immense significance and value. Advanced digital technologies offer new avenues for collecting, processing, storing, and particularly disseminating ICH data, effectively advancing the endeavors of ICH cultural industries and providing diverse means to safeguard and promote ICH culture.

**Fig 1:**
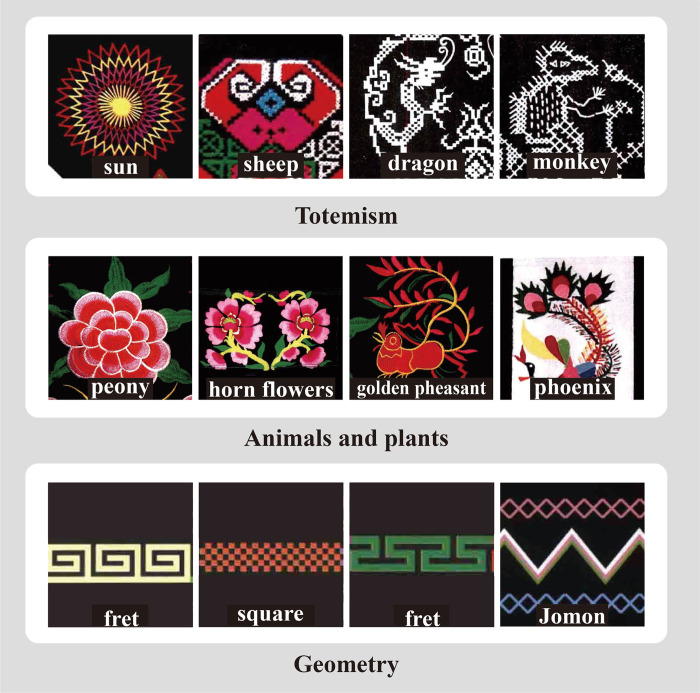
Classification image of typical patterns of Qiang embroidery.

The Qiang ethnic group, an ancient minority in China, primarily resides in the northwest of Sichuan Province. Through their long-standing production practices and daily activities, the Qiang people have created a culturally distinctive art form. Qiang embroidery, a representative cultural expression of the Qiang people, was officially recognized as a national ICH in 2008. The intricate patterns found in Qiang embroidery encapsulate the material and spiritual aspects of the Qiang people’s cultural life, embodying their aesthetic ideals and artistic ingenuity. To be specific, Qiang embroidery patterns derive inspiration from a diverse array of sources, including trees, flowers, fruits, grains, birds, animals, insects, and fish. These patterns encompass the material environment and spiritual beliefs of the Qiang people, commonly found in daily objects, with a predominant presence in clothing decorations. For instance, Qiang embroidery patterns can be categorized into three main groups: totemism, animals and plants motifs, and geometric designs, as illustrated in Fig 1.

Currently, the preservation of Qiang embroidery has encountered certain threats due to various factors, including social development, human activities, environmental changes, and natural disasters. For example, the devastating 2008 Wenchuan earthquake had a profound impact on Qiang ethnic culture, resulting in the destruction of major Qiang settlements and the unfortunate loss of over 80% of cultural inheritors and research experts at the Beichuan Qiang Research Institute. This tragedy also destroyed a substantial amount of materials and electronic data stored within the institute [1]. Hence, this paper explores the state of digital preservation measures by focusing on the stylistic features and modern inheritance dilemma of Qiang embroidery patterns. It elucidates the value of vectorization in digitally preserving Qiang embroidery patterns and underscores the pressing need for novel vectorization techniques in digital image processing for Qiang embroidery. Furthermore, it investigates the feasibility of employing edge detection techniques in the vectorization process of Qiang embroidery patterns. By utilizing the Xception deep learning algorithm for edge detection, this study holds significant implications for the digital preservation and modern inheritance of Qiang embroidery, the support of ICH cultural industry development, and the promotion of relevant ICH cultural database construction [2–5]. In addition, we compared the Xception to the other common edge detection approaches and demonstrated that the Xception achieves the best results. The contributions of this paper includes: (1) The use of the Xception algorithm based on convolutional neural networks effectively solves the problem of extraction the shape of Qiang embroidery in two-dimensional vectorial images, providing a practical solution for processing fine-grained and complex-edge images. (2) It is clearly evident that effective pre-processing techniques play a crucial role in the removal of isolated noise points within blank areas and the enhancement of pixel consistency throughout the entire image. (3) The proposed method offers a reliable practical reference for the preservation of other related intangible cultural heritage images, promoting the application of AI in the field of ICH preservation.

The paper is organized as follows: Section 2 reviews related work on two aspects, the status quo of Qiang embroidery and techniques of image edge detection, and Section 3 describes the Xception model. Our experimental evaluation is presented in Section 4. The conclusion and future work are presented in Section 5.

## 2. Literature review

### 2.1. The status Quo of Qiang embroidery patterns preservation

Since its inclusion in the national ICH list in 2008, Qiang embroidery has garnered attention from local governments and the wider public. In recent years, official efforts to support and promote the inheritance and development of Qiang embroidery have increased. These initiatives encompass the establishment of Qiang ethnic cultural ecological protection experimental zones, the creation of ICH experience centers, and the organization of training programs to disseminate skills related to Qiang embroidery and other ICH practices.

In the digital preservation of Qiang embroidery patterns, digital processing plays a crucial role in various stages, including the presentation of digitized resources such as texts, audio, and videos related to Qiang embroidery in the early stage, the classification and integration of digital resources in the storage phase, and the diversity of digital dissemination and display in the later stage [6,7]. Within the entire digital processing stage, the process of vectorization holds significant importance. Typically, the original image resources of Qiang embroidery patterns are in bitmap format. However, bitmap images have limitations such as resolution constraints, susceptibility to distortion during scaling and editing, large file sizes, and the lack of support for transparency. In contrast, vectorized images obtained through vectorization processing offer several advantages. They possess recognizability, editability, and replicability. Vectorized images allow for rapid identification and searching of specific Qiang embroidery patterns, distortion-free editing and scaling, and occupy less file space. This makes them highly suitable for preservation, application, restoration, or modification purposes.

Vectorized Qiang embroidery patterns can be disassembled into pattern parts as needed, enabling rotation, arrangement, repetition, and combination to achieve various types of storage archiving. This provides resource support for the development and utilization of modern creative Qiang embroidery pattern designs. Furthermore, vectorization meets the requirements of digital dissemination in various forms, such as virtual reality and augmented reality interactive displays, and facilitates the sharing of digital resources related to ICH. It also contributes to the promotion and dissemination of multimedia and multi-platform Qiang embroidery patterns.

Currently, most researchers and scholars rely on software such as Adobe Photoshop and Adobe Illustrator for bitmap processing and vector graphic creation when vectorizing traditional patterns [8–10]. However, the workload of image vectorization is substantial and complex. Moreover, manual drawing work is subjective and may not guarantee a completely objective restoration of Qiang embroidery patterns. Therefore, there is an urgent need for innovation in vector graphic processing technology to ensure the inheritance of Qiang embroidery ICH and related culture, as well as the synchronous development of the industry. This innovation would maximize the savings of human, material, and time resources while achieving the early realization of shared digital cultural resources.

### 2.2. The SOTA of image edge detection

Image edges indicate areas in an image where pixel variations are the most pronounced. They contain valuable inherent information and play a crucial role in extracting image features for image recognition. Edge detection has been a significant research focus since its inception, as it forms a fundamental step in image pre-processing. It serves as a solid foundation for subsequent deep-level image processing and finds applications in various fields such as image segmentation, scene recognition and object detection.

With the advancement of AI algorithms in image processing, CNNs have emerged as a prominent research area, gradually replacing traditional algorithms and achieving remarkable results in edge extraction. However, limited research has been conducted on applying edge extraction techniques to Qiang embroidery. Traditional edge detection algorithms, such as Canny [11], Sobel [12], Roberts operator [13], and Log operator [14], mainly rely on low-level local cues (such as color and texture) for edge extraction. These methods face challenges in accurately representing complex scenes and are often affected by noise and texture clarity, resulting in intermittent and incomplete edge maps. Qiang embroidery patterns are renowned for their intricate details and complex patterns [15]. Experimental findings have indicated that traditional edge extraction algorithms fail to meet the requirements for accurately extracting edges in embroidery patterns. Therefore, it is crucial to enhance traditional algorithms and conduct in-depth research by combining them with CNN methods. For example, Ray etc. [16] introduced an advanced CNN to retain more edge pixels over conventional edge detection algorithms. Inspired by deep learning, in this paper, we aim to extract more high-level semantic information from embroidery images, thereby enhancing the clarity and completeness of the patterns. We would like to harness the power of image edge detection techniques on the Qiang embroidery patterns and explore an innovative idea to preserve such patterns, as well as give empirical practice for other ICH images.

## 3. Methodology

### 3.1. Framework

**Fig 2:**
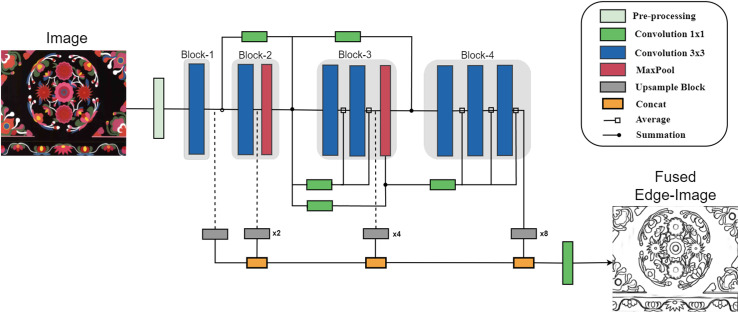
Edge extraction network structure diagram.

The overall structure of our model is presented in Fig 2. In this paper, Qiang ethnic embroidery patterns can be categorized into two groups: digital images and physical images. However, the generation and transmission process of these images may generate noise, leading to degraded image quality, compromised visual effects and obstacles in subsequent processing. Therefore, image pre-processing plays a crucial role in mitigating these issues. The objective of pre-processing is to smoothen image edges, eliminate jaggedness, reduce noise interference caused by uncertain factors like photography, and enhance image contrast. This process facilitates the extraction of continuous edge contours during edge detection. To optimize the images for edge extraction, we first employ specific pre-processing techniques tailored to different scenes, aiming to enhance the visual effects of the images. These techniques transform the images into a format feature that is more suitable for machine analysis. The process involves selectively emphasizing meaningful information for analysis, suppressing irrelevant details, and maximizing the value of image utilization. Subsequently, the CNN is employed to extract edges from the pre-processed images. Finally, the obtained edge maps undergo edge vectorization using the online conversion tool Autotrace and output the scalable vector graphics(SVG) images. The individual modules will be described in detail later.

### 3.2. Edge extraction neural network based on Xception

**Fig 3:**
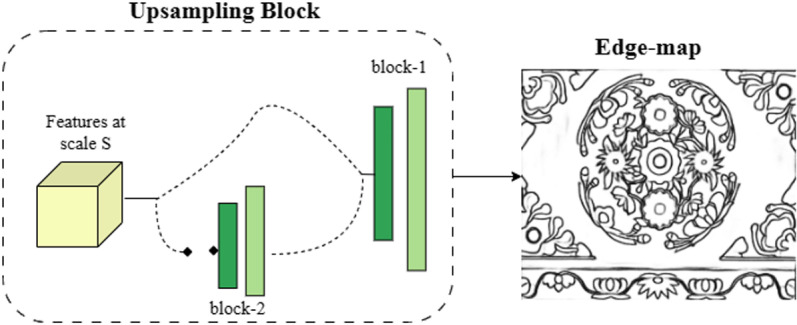
Upsampling network model architecture diagram.

The model in this paper is trained end-to-end, eliminating the need for weight initialization from pre-processing detection models, which is typically required by most deep learning-based edge detectors. However, the deep learning model with a number of layers faces the problem of vanishing gradient. To address the issue of edge features being lost in deeper layers, an Xception-based architecture is adopted in this study. This architecture utilizes parallel connections to capture edge information across different layers. Considering the substantial amount of information contained in images, batch processing of images can significantly increase computational costs. Therefore, factors such as model performance, complexity, computational resources, and data size are taken into account when selecting a model for this paper. A model with fewer than 0.7 million parameters is chosen to strike a balance, ensuring ease of use and deployment in resource-constrained environments while maintaining effective edge extraction. The overall network architecture of the model can be visualized as an edge extraction network with an upsampling sub-network, as illustrated in Figs 2 and 3. The edge extraction structure takes an image as input, undergoes pre-processing, and then passes through different blocks for convolutional processing.

The edge extraction network comprises four output blocks (Block-1 to Block-4), drawing inspiration from the Xception network. Each block consists of sub-blocks containing convolutional layers, and parallel connections connect the blocks and sub-blocks. Each sub-block consists of a stack of two convolutional layers, followed by batch normalization and the ReLU activation function (except for the last convolutional layer in the final sub-block, which lacks this activation).

Due to the numerous convolutions performed, crucial edge features can be lost within each deep block, rendering a single main connection insufficient. To address this, starting from Block-3, the output of each sub-block is averaged using edge connections before being combined with the main connection, as depicted in Fig 2. Following the max pooling operation, the edge connections are configured to average the output of each sub-block. The feature maps generated at each block are then fed back into a separate upsampling network, producing intermediate edge maps. These intermediate edge maps are concatenated to form a stack of learned filters. Finally, at the end of the model, these features are fused into a single edge map.

The upsampling process consists of a conditional stack comprising two blocks (Block-1 and Block-2). Each block consists of a sequence involving a convolutional layer followed by a transpose convolutional layer. Block-1 handles the input with a 1 × 1 kernel size and applies a ReLU activation function. The transpose convolution in Block-1 utilizes a kernel size of s × s, where s represents the scale level of the input feature map. Block-2 is activated when scaling the input feature maps from the initial network. Once this condition is met, the feature map is fed back to Block-1.

### 3.3. Loss function

A set of predicted edge is obtained as output of $l_{txs}$. Therefore, the resulting loss function is as follows: $\hat{Y}\in R^{m\times n\times 1}$ from the given RGB image $X\in R^{m\times n\times 3}$ (where *m* and *n* represent the size of the image), and the edge output are evaluated using the corresponding ground truth values*Y*, ${\hat{Y}}_{P}$ denotes the final output of the model, which is generated through data fusion from the initial network. The overall loss function *l*, is applied to each intermediate edge output ${\hat{Y}}_{i}$(i = 1, 2, 3, 4). It consists of tracing loss $l_{t}$, boundary tracing loss $l_{bt}$, and texture suppression loss


$$l=l_{t}+\alpha_{bt}\times l_{bt}+\alpha_{txs}\times l_{txs}$$ (1)

Where $\alpha_{bt}$ is the weight for the regularized edge loss, $\alpha_{txs}$ is the loss for suppressing texture in each prediction. The final loss is the sum of the losses predicted by each sub-block. The cross-entropy loss $l_{t}$ is defined as follows:


$$l_{t}\left({\hat{Y}}_{i},Y\right)=1/\left(m\times n\right)-w\left(Y\cdot \log{\hat{Y}}_{i}+\left(1-Y\right)\cdot \log\left(1-{\hat{Y}}_{i}\right)\right)$$ (2)

$$w\left(Y=1\right)=1\text{*}\frac{Y_{-}}{Y_{+}+Y_{-}};w\left(Y=0\right)=1.1\text{*}\frac{Y_{+}}{Y_{+}+Y_{-}};w\left(Y=2\right)=0$$ (3)

Where *w* is the loss weight, and $Y_{+}$, $Y_{-}$ represent the positive and negative edge samples in the given ground truth, respectively. Regarding the boundary tracing loss $l_{bt}$, it is defined as follows:


$$l_{bt}\left({\hat{Y}}_{i},Y\right)=1/\left(m\times n\right)-\underset{p\in E}{\sum }\log\left(\underset{j\in D_{p}}{\sum }\frac{{\hat{Y}}_{j}}{\sum_{j\in R_{p}\backslash D_{p}}{\hat{Y}}_{j}+\sum_{j\in D_{p}}{\hat{Y}}_{j}}\right)$$ (4)

Where *E* is the given reference value, $R_{p}$ represents the edge map centered at non-edge point *p*, $D_{p}$ is the center point of edge $R_{p}$ and *j* takes values 1, 2, 3, 4. For the texture suppression loss $l_{txs}$, it is defined as follows:


$$l_{txs}\left({\hat{Y}}_{i},Y\right)=1/\left(m\times n\right)-\underset{p\in Y\backslash \hat{E}}{\sum }\log\left(1-\underset{j\in R_{p}^{t}}{\sum }\left({\hat{Y}}_{i}^{j}\right)/\left|R_{p}^{t}\right|\right)$$ (5)

Where $\hat{E}$ is the set that includes all edges and their confounding pixels used in the boundary loss function. serves as a buffer to reduce negative interactions between weak edges and texture regions.

## 4. Experiments and results

### 4.1. Data pre-processing

Three datasets have been used for training the proposed model: MDBD [17], BIPED [18], and BRIND [19]. We directly use the pre-trained model for our experiments due to the limited size of training data. We collected the Qiang embroidery patterns data from the Internet, the datasize is 33. Considering the limited availability of texture samples in the application scenario and the effectiveness of traditional denoising methods, such as mean filtering, median filtering, and Gaussian filtering [20,21], we have employed these methods in our study. However, spatial domain denoising techniques often involve a trade-off between noise removal and preserving image details. We have utilized IAMF and non-local mean filtering for denoising purposes on the two types of images respectively. After pre-processing of the image, the color original is converted into a denoised grayscale image for input to the model.

**Fig 4:**
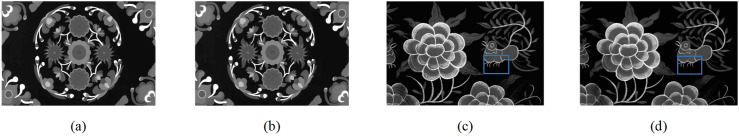
Pre-processing Result Graph for Both Types of Images: (a) Digital Image; (b) Diagram of the processing result of the IAMF method; (c) Physical Image; (d) Diagram of the processing result of the Non-local mean method.

We adapt IAMF and select the sliding window sizes based on the spatial correlation principle of image processing. As the window size increases to its maximum value in the algorithm, IAMF effectively removes noise while preserving fine details in the image edges. For physical image, we chose non-local mean filtering to denoise, it calculates weights based on similarity measures and leverages redundant information in the image. This approach not only reduces noise but also preserves detailed image features to the maximum extent. In Fig 4, we provide two examples of pre-processed images by the use of different pre-processing approaches, which shows the effects of the employed denoising techniques. For both digital images and physical images, choosing a specific denoising approach would improve the quality of feature extraction.

**Fig 5:**
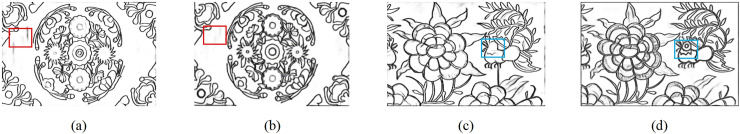
Comparison of Edges with and without Pre-processing: (a) Pre-processing edge of Digital Image; (b) Unpre-processing edge of Digital Image; (c) Pre-processing edge of Physical Image; (d) Unpre-processing edge of Physical Image.

In addition, it can be observed that the pre-processing edge map reduces noise in the low saturation regions, as shown in Fig 5 (a) and (b), enhancing the continuity and visibility of the texture edge map. It effectively reduces the loss of some isolated edges, as seen in Fig 5 (c) and (d), making the information of texture edges more abundant.

### 4.2. Edge extraction

**Fig 6:**
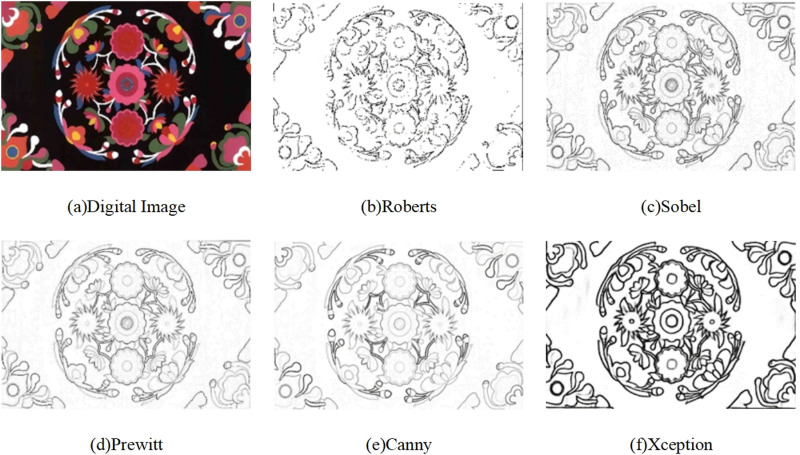
Digital Images Comparison Experiment.

**Fig 7:**
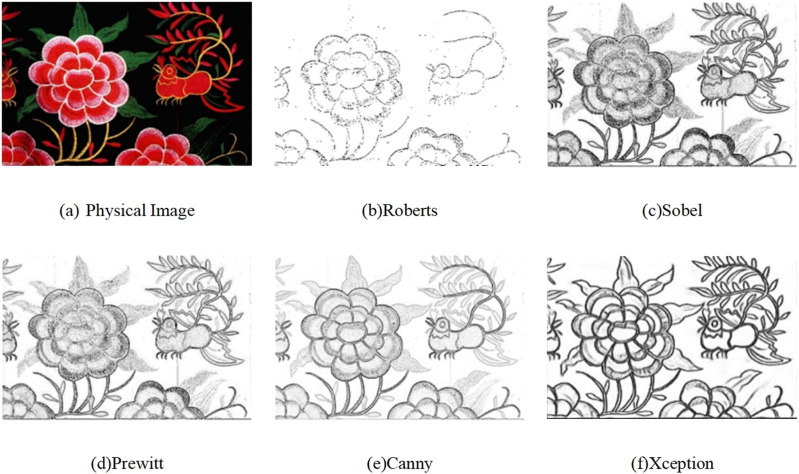
Physical Images Comparison Experiment.

In this study, we utilized the Xception deep learning model for edge extraction of Qiang embroidery patterns. As a comparative analysis, we conducted reference experiments using traditional edge detection operators, specifically Roberts, Sobel, Prewitt, and Canny, on both digital images and physical images, as depicted in Figs 6 and 7.

From the aforementioned figures, it can be observed that the Roberts operator exhibits sensitivity to noise, rendering it ineffective in eliminating local interference. Furthermore, it struggles to detect and identify weak edges characterized by subtle grayscale differences between the target and the background, resulting in fragmented extracted edges. Additionally, manual threshold setting is required for this operator, limiting its efficacy in extracting various object contours.

The Sobel operator performs well in processing images with grayscale gradients and high noise levels. However, it relies on directional templates for edge extraction, making it less suitable for accurately capturing contours in images with complex textures and diagonal edges. The Prewitt operator shares similar principles and effects with the Sobel operator, but it may yield incomplete edge detection outcomes. Conversely, the Canny operator exhibits reduced susceptibility to noise interference and demonstrates the ability to detect faint edges. Nevertheless, it is sensitive to gradient calculations and necessitates manual adjustment of the Gaussian filter’s variance, potentially leading to missed detections.

To assess the efficacy of edge enhancement in images, we employed two evaluation metrics: image entropy H [22–24] and structural similarity index (SSIM) [25–27]. In Equation 6, $P_{i}$ denotes the probability density of grey levels; Lis the maximum grey level. Image entropy H quantifies the level of uniformity in grayscale values within an image, with higher values indicating a larger amount of information present. On the other hand, SSIM, ranging from 0 to 1, gauges the preservation of structural and depth information in the image, with higher values indicating better preservation. $U_{x}$ and $U_{y}$ denote the luminance mean of image J and image GJ, respectively, in Equation 7. $\sigma_{x}$ and $\sigma_{y}$ denote the standard deviation of image J and image GJ, respectively; $c_{1}$ and $c_{2}$ are parameter constants. The detailed evaluation results are presented in Tables 1 and 2.

**Table 1:** The comparison data table for digital image.

Methods	H	SSIM
Rober	0.3774	0.0396
Sobel	4.5286	0.1150
Prewitt	4.6063	0.1056
Canny	3.7041	0.1085
Xception (Ours)	**4.8705**	**0.2681**

**Table 2:** The comparison data table for physical image.

Methods	H	SSIM
Rober	0.3774	0.0221
Sobel	5.5312	0.2099
Prewitt	5.6966	0.1876
Canny	5.0554	0.1624
Xception (Ours)	5.7373	0.3599


$$H=-\sum_{i=0}^{L}P_{i}\log_{2}P_{i}$$ (6)

$$SSIM=\frac{\left(2u_{x}u_{y}+c_{1})(2\sigma_{x}\sigma_{y}+u_{2}\right)}{\left(u_{x}+u_{y}+c_{1})(\sigma_{x}^{2}+\sigma_{y}^{2}+c_{2}\right)}$$ (7)

Based on the presented tables, it is evident that the Xception exhibits superior performance compared to other traditional algorithms. It not only yields higher image entropy values but also achieves a minimum 0.1 increase in the structural similarity index (SSIM) compared to the alternative methods. Remarkably, for the physical image, the Xception surpasses the Roberts operator by 0.3, clearly demonstrating its superiority in this specific domain. Based on the Friedman test (p value = 0.05), we provided that the improvement of Xceptions is statistical significant comparing to the other methods. The effectiveness of the Xception can be attributed to the optimization of the loss function and the incorporation of information fusion operations. These enhancements enable the Xception to extract detailed information while effectively representing the overall structural characteristics of the images.

### 4.3. Deep learning methods

In the comparative experiments, we implemented the HED (Holistically-Nested Edge Detection) algorithm based on the VGG16 network [28]. HED utilizes a multi-scale network architecture that combines feature maps of different resolutions, ranging from fine to coarse. This design allows the network to capture edge information at various scales, including both subtle edge details and broad structural edges, thereby enhancing the accuracy and robustness of edge detection. The purpose of this comparison is to validate the advantages of the Xception algorithm over other deep learning algorithms in extracting image edge information, particularly concerning edge accuracy and detail preservation.

**Fig 8:**
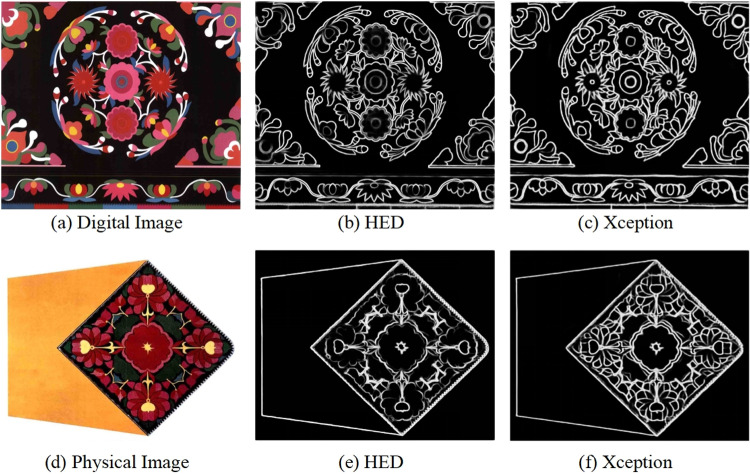
Comparison Experiment with deep learning methods.

Fig 8 presents a comparison of the edge detection results of the two algorithms on two representative images. The first digital image contains rich texture details and a complex background, while the second real-world image focuses on the object’s contours and fine structures. As illustrated, although the HED algorithm performs well in overall contour detection, it exhibits some breakages and discontinuities when processing fine edges and texture regions, leading to less-than-ideal edge continuity. In contrast, the algorithm based on the Xception architecture not only maintains edge continuity but also better captures the fine edges and texture details of the images. The edge lines are smoother and more precise, especially at the junctions between object edges and complex backgrounds, where its performance is particularly outstanding.

To further quantitatively evaluate the performance of the two algorithms, we adopted the Pratt Figure of Merit (FOM) [29] as the evaluation metric. This metric is primarily used to assess the agreement between the edge detection results and the ideal edges, comprehensively considering the accuracy, continuity, and localization precision of edge detection. The Pratt Figure of Merit is defined as:


$$FOM=\frac{1}{N_{e}}\overset{N_{e}}{\underset{i=1}{\sum }}e^{-\alpha d_{i}^{2}}$$ (8)

where $N_{e}$ is the number of predicted edge pixels, $d_{i}$ is the distance from the ith predicted edge pixel to the nearest true edge pixel, and α is a positive coefficient to adjust the degree of influence of distance.

As shown in Table 3, for both test images, the Xception algorithm significantly outperforms the HED algorithm in terms of the Pratt metric, with improvements exceeding 10%. This result verifies the superiority of the Xception algorithm in edge detection tasks. This advantage is manifested not only in the overall accuracy of edge detection but also in the precise capture and preservation of fine edge details.

**Table 3:** Comparison of FOM metrics for the two types of images.

Methods	(a) Digital Image	(d) Physical Image
HED	0.2522	0.0886
Xception (Ours)	**0.3253**	**0.0967**

### 4.4. Edge vectorization

**Fig 9:**
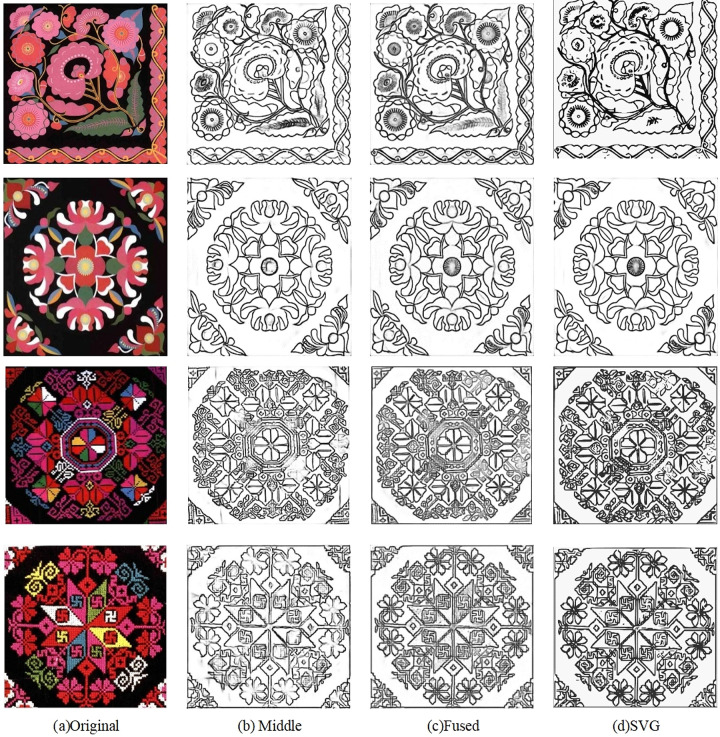
The result of Edge Extraction of Qiang Embroidery Images.

The trained network model was employed to extract the edges from Qiang embroidery patterns. Fig 9(a) showcases some example Qiang embroidery patterns, while Fig 9(b) exhibits the edge fusion image generated by the initial network. The experimental result is depicted in Fig 9(c), and subsequently, the vectorized image is obtained using an online vectorization tool, as demonstrated in Fig 9(d).

Based on the experimental results presented in Figs 6–9, it is evident that effective pre-processing techniques contribute to the removal of isolated noise points in blank areas and the enhancement of pixel consistency throughout the image. Training the edge extraction model using the entire image enables the acquisition of rich high-level semantic information. When this information is combined with low-level feature information, the fused edge image demonstrates improved edge continuity and smoothness. Consequently, the generated vectorized image successfully meets the quality requirements for accurately depicting Qiang embroidery patterns.

### 4.5. Analysis of results

Given the diverse range of Qiang embroidery patterns encountered in daily life, traditional edge detection methods may not be suitable for all types of images. To address this challenge, we adopted an edge detection approach based on the Xception deep learning algorithm. In this study, we conducted experiments on a sequence of images, including digital images (Group (a): images 1 and 2) and physical images (images 3 and 4). As depicted in Fig 9, after applying distinct pre-processing techniques to each image type, notable observations can be made. The fused edge images in Group (b) exhibit edge aliasing and a loss of details in less prominent areas. However, after merging the intermediate edge maps, the final edge images in Group (c) showcase enhanced edge connectivity, effectively minimizing edge fragmentation and misidentification. By converting the resulting edge images into SVG format using an online conversion tool, they can be infinitely scaled, satisfying the quality requirements for accurately depicting Qiang embroidery patterns. These findings carry substantial academic research significance and offer practical value in digitally preserving Qiang embroidery patterns, such as for Qiang cultural promotion posters, brochures, and other tourism-related materials.

## 5. Conclusions

This paper applies the popular deep learning neural network named Xception to perform vectorized edge extraction on Qiang embroidery patterns. The visual results clearly demonstrate the effectiveness of this approach in addressing the challenging task of vectorizing Qiang embroidery and related ICH two-dimensional images. By utilizing this method, the wider dissemination of Qiang embroidery patterns is facilitated, thereby contributing to the inheritance and development of Qiang embroidery culture. The vectorized Qiang embroidery patterns can be applied and promoted in various dimensions:

(1) Educational and entertaining preservation of ICH: The Qiang embroidery patterns extracted through edge detection techniques accurately depict the complete edges and internal contours of the patterns, resembling line drawings. These patterns serve as excellent teaching materials for conveying the visual characteristics and meanings of Qiang embroidery. By integrating ethnic culture into coloring books, these cultural elements can be introduced to school-age children, offering both educational and entertaining experiences. Moreover, it can serve as a stress-relieving activity for adults. Due to their vectorized nature, these patterns can be applied not only in traditional paper media but also in new media platforms such as mobile apps.
(2) Innovative reconstruction design incorporating modern styles: By deconstructing the vectorized Qiang embroidery patterns and applying transformations like rotation and scaling, they can be reconstructed in line with modern aesthetic trends. This enables the creation of innovative graphics that embody traditional aesthetic qualities while meeting the demands of contemporary society. These designs can be applied in various cultural and creative derivative products, including fashion design, product packaging, decorative accessories, handicrafts, and more.
(3) Cultural promotion through digital media design: Diverse digital media technologies provide new opportunities for the public to gain in-depth knowledge of Chinese ICH The rich foundation of vectorized data can be integrated into emerging cultural and technological formats. It is anticipated that Qiang embroidery will embrace technologies such as holographic projection, virtual reality, augmented reality, etc., to present this intangible art form in a more complete, three-dimensional, vivid, and engaging manner. This will generate public interest and enthusiasm for learning Qiang embroidery, creating new opportunities for its development and survival.

The utilization of edge detection techniques for rapid vectorization of Qiang embroidery patterns not only increases their recognition and acceptance among the general public but also offers new insights for updating vectorization techniques and applying their outcomes to communication and innovative design in other domains of traditional culture and intangible heritage.

## Supporting information

S1 File XED. (ZIP)
