# Supplementary figures and images for: Generate vector graphics of fine-grained pattern based on the Xception edge detection
Source: PLoS One. 2025 Jun 11;20(6):e0318930. doi: 10.1371/journal.pone.0318930 (PMC12157116; doi:10.1371/journal.pone.0318930)

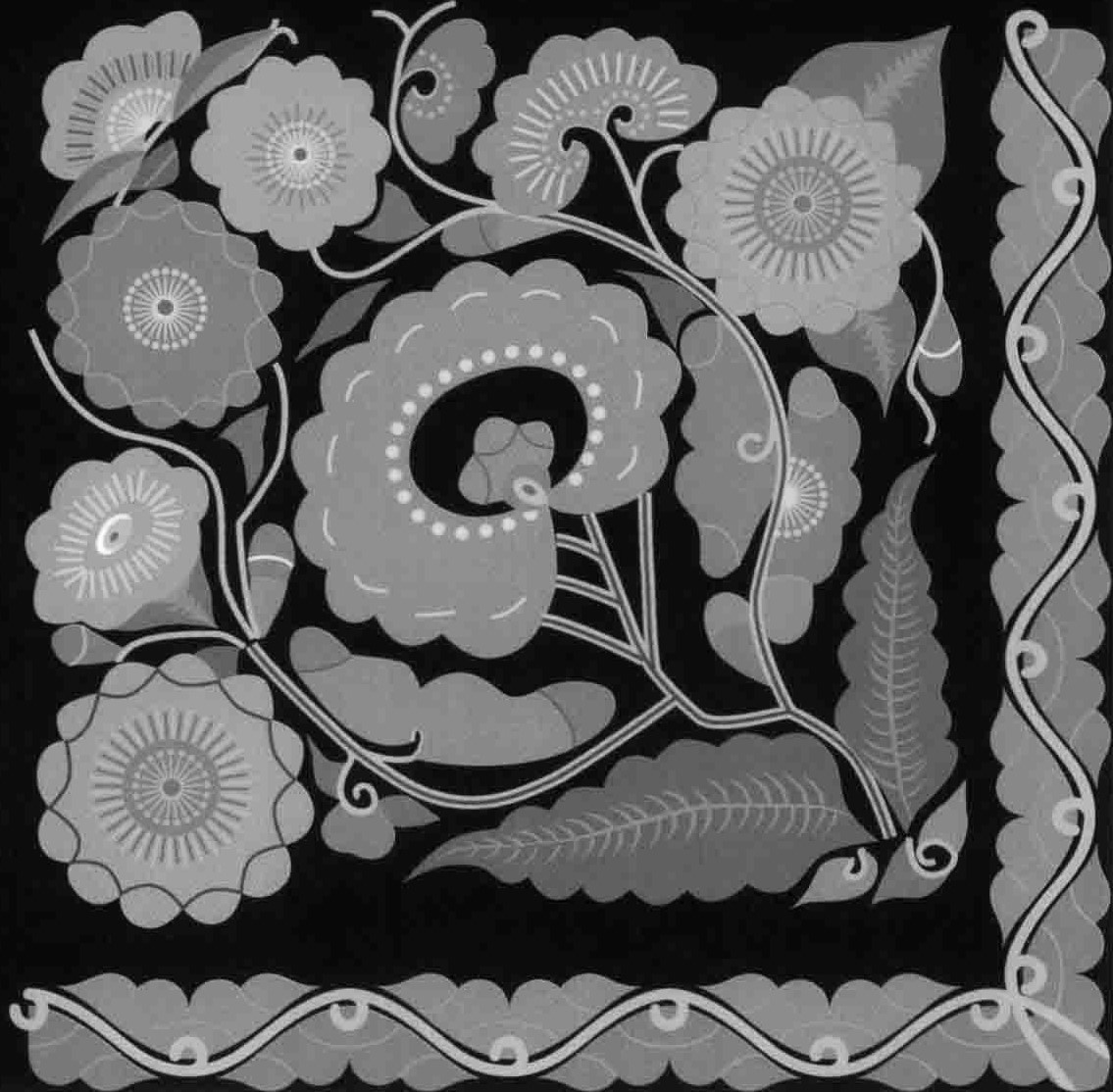

Supplement: S1 File — (ZIP) [file pone.0318930.s001.zip › XED/data/178.jpg]

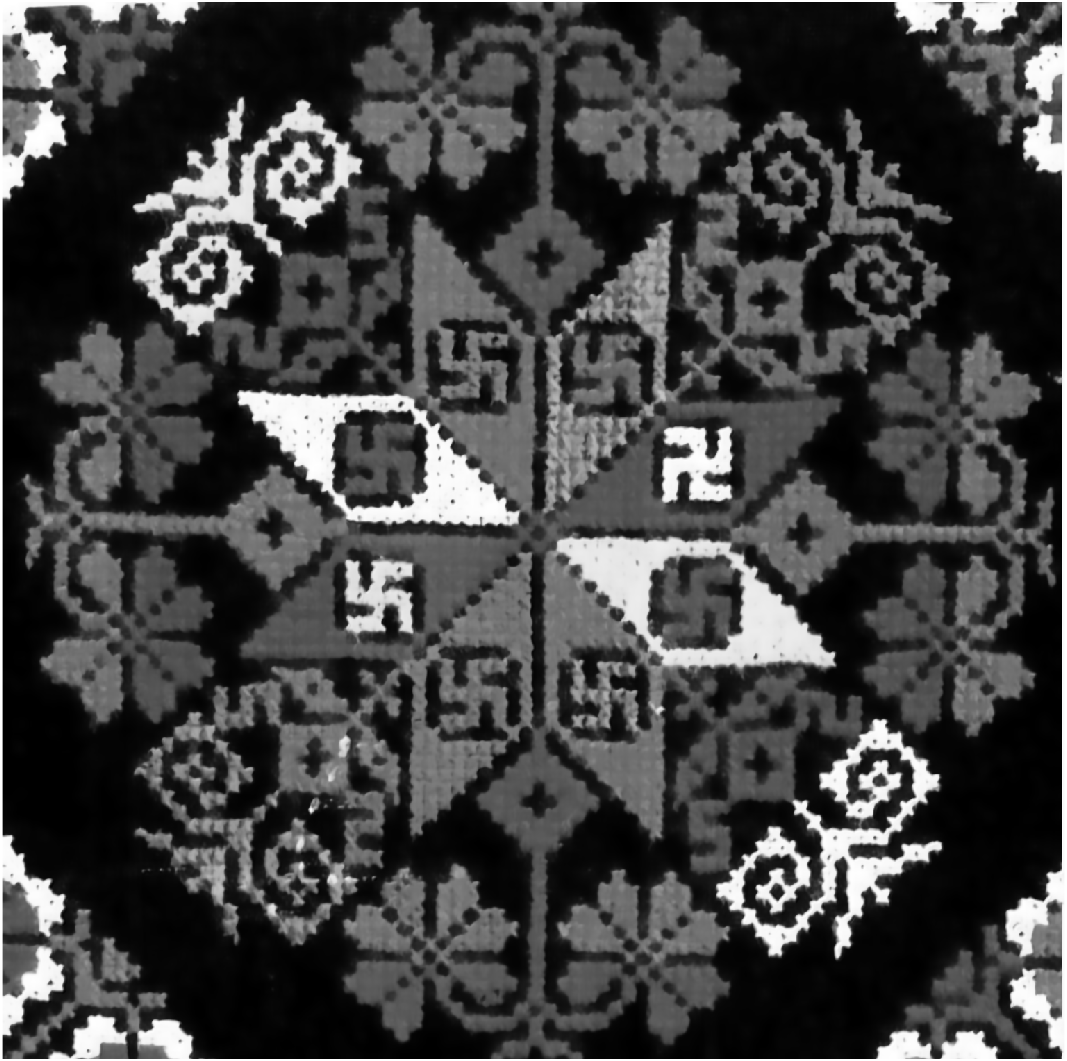

Supplement: S1 File — (ZIP) [file pone.0318930.s001.zip › XED/data/fjb1.png]

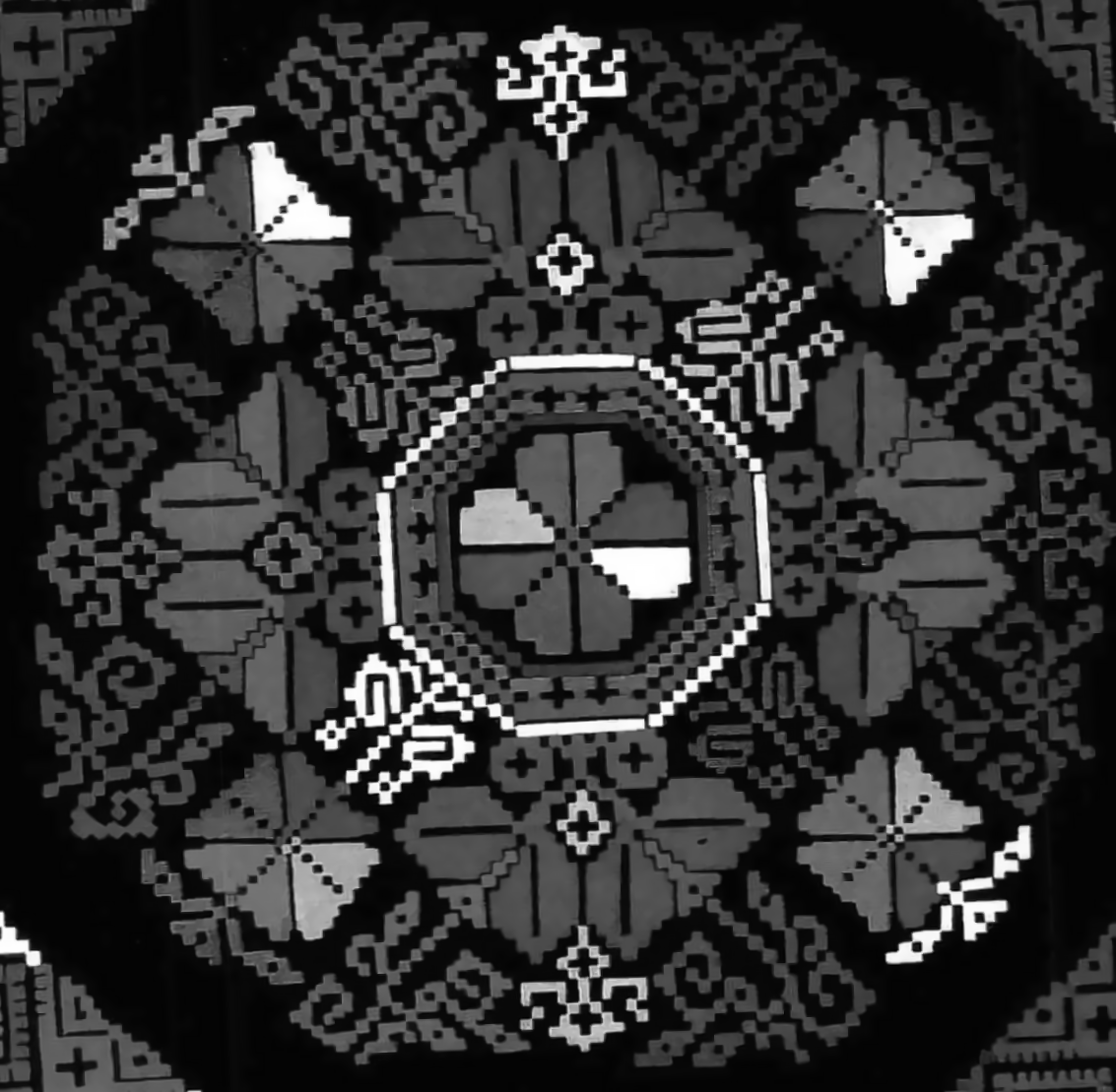

Supplement: S1 File — (ZIP) [file pone.0318930.s001.zip › XED/data/result1.png]
